# FANCD2 inhibits ferroptosis by regulating the JAK2/STAT3 pathway in osteosarcoma

**DOI:** 10.1186/s12885-023-10626-7

**Published:** 2023-02-22

**Authors:** Xujun Li, Jiangyi Liu

**Affiliations:** Department of Orthopaedic, Minhang Hospital, Fudan University, No.170, Xinsong Road, Xinzhuang Town, Minhang District, Shanghai City, 201199 China

**Keywords:** FANCD2, Ferroptosis, JAK2/STAT3 pathway, Osteosarcoma

## Abstract

**Background:**

This research aimed to investigate the roles of fanconi anemia complementation group D2 (FANCD2) on the regulation of ferroptosis in osteosarcoma progression.

**Methods:**

The function of FANCD2 on cell viability, invasion, migration, and tumor growth were explored. FANCD2 and pathway-related genes were determined by western blot. Ferroptosis-associated markers were determined, including lipid peroxidation, labile iron pool (LIP), ferrous iron (Fe^2+^), and ferroptosis-related genes.

**Results:**

FANCD2 expression was increased in osteosarcoma cells. FANCD2 knockdown reduced cell viability, invasion, and migration of osteosarcoma cells. FANCD2 knockdown regulated ferroptosis-related gene expression, and distinctly increased the levels of LIP, Fe^2+^, and lipid peroxidation, and these effects were reversed by a ferroptosis inhibitor Fer-1. In addition, JAK2 and STAT3 expression were reduced by silencing of FANCD2, and STAT3 activator (colivelin) distinctly reversed tumor suppressor effects of FANCD2 silencing on osteosarcoma development.

**Conclusion:**

These findings suggested that FANCD2 silencing could suppress osteosarcoma cell viability, migration, invasion, and tumor growth, and induced ferroptosis by regulating the JAK2/STAT3 axis. These findings may provide novel therapeutic ideas for clinical treatment of osteosarcoma.

**Supplementary Information:**

The online version contains supplementary material available at 10.1186/s12885-023-10626-7.

## Introduction

Osteosarcoma is a most common aggressive bone tumor, which usually derives from malignant mesenchymal cells of bone [1]. Osteosarcoma mostly occurs in adolescents, the five-year survival of osteosarcoma patients is about 70% [2], surgery and adjuvant chemotherapy are common treatments for osteosarcoma [3, 4]. Due to early metastasis and the development of chemoresistance, the survival of osteosarcoma is unsatisfactory [5]. Thus, it is urgently needed to find potential treatment targets to improve prognosis.

Fanconi anemia complementation group D2 (FANCD2) is involved in the regulation of tumorigenesis, apoptosis, and other life processes in cancers [6], such as glioblastoma [7], esophageal squamous cell carcinoma (ESCC) [8], and lung adenocarcinoma [9]. Elevated FANCD2 expression is correlated with a poor prognosis in primary and recurrent glioblastoma, silencing of FANCD2 inhibits cell survival [7]. Inhibition of FANCD2 distinctly inhibits cell proliferation, metastasis, and cell cycle progression in ESCC [8]. FANCD2 can predict the survival, tumor immunity, chemotherapy sensitivity, and mutation burden of lung adenocarcinoma [9]. Furthermore, the reduction of FANCD2 expression promotes caspase-dependent cell apoptosis by phosphorylation of p53 in osteosarcoma cells (MG-63) [6]. However, the detailed roles of FANCD2 in osteosarcoma development are not fully clear.

Ferroptosis is a novel type of iron-dependent cell death that is driven by a fatal increase in lipid peroxidation [10]. The inhibition of glutathione peroxidase 4 (GPX4) can disrupt intracellular iron homeostasis and suppress lipid peroxide reducibility to induce ferroptosis, thereby inhibiting the proliferation and tumor growth of ovarian cancer [11]. Ferroptosis exerts an essential role in osteosarcoma progression and target ferroptosis may be an effective therapeutic strategy [4]. Bavachin inhibits the viability of MG63 and HOS cells, increases the levels of Fe^2+^ and ROS accumulation, promotes malondialdehyde, and reduces glutathione to induce ferroptosis, thereby exerting anti-tumor effects on osteosarcoma [12]. Furthermore, in bone marrow stromal cells, FANCD2 involves in the regulation of genes associated with iron metabolism (such as FTH1 and TFRC) and lipid peroxidation (such as GPX4) [13]. FANCD2 promotes temozolomide resistance by slowing ferroptosis in glioblastoma cells [7]. However, the role of FANCD2 in regulating ferroptosis of osteosarcoma cells remains unclear.

Some studies have demonstrated that the Janus-activated kinase 2 (JAK2)/signal transducer and activator of transcription 3 (STAT3) pathway are correlated with tumor progression, such as gastric cancer [14], colorectal cancer [15], and hepatocellular carcinoma [16]. Previous research has indicated that piperlongumine inhibits the JAK2/STAT3 pathway to suppress osteosarcoma progression [17]. However, it is unclear on the relationship between JAK2/STAT3 pathway and FANCD2 in osteosarcoma.

In the present study, we investigated the function of FANCD2 silencing on osteosarcoma cells, tumor growth, and ferroptosis. Furthermore, the interaction between FANCD2 and JAK2/STAT3 pathway was further explored. Our results may FANCD2 may provide a novel marker for osteosarcoma and a potential therapeutic strategy for clinical treatment.

## Materials and methods

### Cell culture and transfection

The human osteosarcoma cell lines (MG-63 and U2OS) and osteoblast cells (hFOB1.19) were obtained from Chinese Academy of Sciences Cell Bank (Shanghai, China), and cultured in DMEM medium (Invitrogen, Carlsbad, CA, USA) containing 10% FBS, 1% streptomycin/penicillin at 37 °C in an atmosphere of 5% CO_2_. siRNA targeting FANCD2 (si-FANCD2) and si-NC were purchased from RiboBio (Shanghai, China). According to the instructions of manufacturer, si-FANCD2 or si-NC were transfected with osteosarcoma cells using Lipofectamine 3000 reagent (Invitrogen) at 37 °C for 48 h. For ferroptosis induction or inhibition, U2OS cells (1 × 10^6^ cells) were further treated with Erastin (10 µΜ, Selleck, Houston, TX, USA) or Fer-1(60 nM, Selleck) for 24 h, respectively. For JAK2/STAT3 signaling pathway activation, cells were treated with colivelin (1 nM, Santa Cruz, Dallas, TX, USA) for 24 h.

### Western blot assay

Osteosarcoma cells were lysed with lysis buffer (Sigma-Aldrich) to extract protein samples after cells were centrifuged. The proteins were separated on 10% SDS-PAGE and transferred to the PVDF membranes. The membranes containing the target protein were cut within a certain range, blocked at 25 °C for 1 h by using 5% skim milk and then incubated with the primary antibodies against FANCD2 (ab108928, 1:1000, Abcam), JAK2 (ab39636, 1:1000, Abcam), p-JAK2 (ab195055, 1:1000, Abcam), STAT3 (ab68153, 1:1000, Abcam), and p-STAT3 (ab76315, 1:2000, Abcam) at 4 °C overnight. Afterward, the membranes were washed and incubated with secondary antibody (1:5000) conjugated by HRP for 1 h at 25 °C. GAPDH was employed for protein loading control. Proteins were visualized using the enhanced chemiluminescence western blotting detection kits (Sigma-Aldrich) on an imaging system (Bio-Rad, CA, USA).

### Quantitative real-time PCR (qRT-PCR)

According to the instructions of manufacturer, total RNA from MG-63 and U2OS cells was extracted using TRIzol reagent (Sigma-Aldrich). Total RNA was reverse-transcribed to CDNA using a Reverse Transcriptase kit (Thermo Scientific, Waltham, MA, USA). RT-qPCR was performed with SYBR® Green Master Mix Kit (Thermo Scientific) and the Mastercycler ep realplex detection system (Eppendorf, Hamburg, Germany). The relative mRNA expression was normalized to β-actin by using the 2^−ΔΔCt^ method. Primer sequences are shown in Table 1.

**Table 1:** Primers for qRT-PCR in this study

Gene	Sequence from 5’-3’
FANCD2 Forward	ACATACCTCGACTCATTGTCAGT
FANCD2 Reverse	TCGGAGGCTTGAAAGGACATC
FTH1 Forward	CGCCAGAACTACCACCAG
FTH1 Reverse	TTCAAAGCCACATCATCG
GPX4 Forward	GAAGCAGGAGCCAGGGAGT
GPX4 Reverse	ACGCAGCCGTTCTTGTCG
COX2 Forward	TGGAGCACCATTCTCCTTGAAAGGACTTAT
COX2 Reverse	GACTGTTTTAATGAGCTCTGGATCTGGAAC
GAPDH Forward	GAATTCATGTTTGAGACCTTCAA
GAPDH Reverse	CCGGATCCATCTCTTGCTCGAAGTCCA

### Cell viability assay

Cell viability of MG-63 and U2OS cells was assessed by using a CCK-8 kit (Thermo Scientific). Osteosarcoma cells were sown in 96-well plates (5 × 10^3^ cells/well) for 24 h at 37 °C. Then, the wells were filled with a 10 µL CCK-8 solution. The OD value (450 nm) was determined by a BioTek microplate reader (Gene Co., Ltd., Shanghai, China) at 24, 48, and 72 h, respectively.

### Wound healing assay

MG-63 and U2OS cells (at a density of 1 × 10^6^ cells) were plated into 6-well plates for 24 h. Sterile plastic tips (200 µL) are used to scrape cells and create interstitial spaces. After cultured for 24 h in reduced serum RPMI 1640 medium, the scratch was observed and images were instantly taken by the Olympus microscope.

### Transwell assay

The invasion of MG-63 and U2OS cells was evaluated by transwell (8 μm pore, Corning, Inc.). The upper surface of the transwell chambers was pre-coated with Matrigel (BD Biosciences, Sparks, USA). The lower chamber was added a complete medium. Cells (2 × 10^5^ cells) were added into the upper chambers with serum-free medium and incubated at 37 °C for 24 h. In the lower chamber, cells were fixed with 4% paraformaldehyde and stained with 0.1% crystal violet for 10 min at 25 °C. Then, the invasion cells were counted by the light microscope.

### Cell proliferation assay

According to the instructions of manufacturer, the proliferation of MG-63 and U2OS cells was detected by the 5-Ethynyl-20-deoxyuridine (EdU) kit (Ribobio, Guangzhou, China). In brief, the treated cells (1 × 10^4^) were seeded into 96-well plates, and then incubated with 50 µM freshly prepared EdU medium for 2 h. Subsequently, cells were fixed with 4% paraformaldehyde for 30 min, decolorized with 2 mg/mL glycine for 5 min, and infiltrated with 0.5% TritonX-100 for 10 min. Then, cells were incubated with Apollo 488 staining solution for 30 min, and counterstained with DAPI (1 mg/ml) for 5 min in the dark. The images were observed under a fluorescence microscope (Leica, Germany), and the EdU-positive rate was calculated.

### Enzyme-linked immunosorbent assay (ELISA)

The levels of SOD, catalase (CAT), and malondialdehyde (MDA) were detected by ELISA assay. The content of SOD, CAT, and MDA was measured using the corresponding detection kit (Esebio, Shanghai, China) according to the instructions.

### Labile iron pool (LIP) and ferrous iron assays

LIP was detected based on the calcein-acetoxymethyl ester method. osteosarcoma cells were treated with calcein acetoxymethyl ester (2 µM) (Corning Inc., Corning, NY, USA) at 37 °C for 30 min, then washed with hanks balanced salt solution. The final concentration of 100 µM deferoxamine mesylate is used to remove the iron in calcein. Then, the fluorescence at 485 nm excitation and 535 nm emissions was detected by the fluorescence plate reader (Thermo Scientific). The fluorescence change was used as an indirect measurement of LIP after the addition of deferoxamine. According to the manufacturer’s instructions, intracellular ferrous iron (Fe^2+^) and iron were measured by an iron assay kit (Abcam), and OD value was measured at 593 nm.

### Animals

Thirty BALB/c nude mice (20 ~ 22 g, five weeks) were SiPeiFu Biotechnology Co., Ltd. (Beijing, China). Animal experiment was conducted in accordance with China Animal Welfare Legislation and was approved by the Ethics Committee of our hospital. U2OS cells (1 × 10^6^ cells) were subcutaneously injected into mice to establish osteosarcoma mice models. The mice were randomized into Lv-si-NC and Lv-si-FANCD2 groups (n = 6). After modeling, lentivirus-packaged si-FANCD2-1 and si-NC were locally injected into tumor tissues of mice in the Lv-si-FANCD2 and Lv-si-NC groups, respectively. They were euthanized by intraperitoneal injection of sodium pentobarbital (50 mg/kg) after 28 days, the tumor tissues were collected and weight was measured. Tumor volume was calculated every seven days using the formula (length × width^2^)/2.

### Immunohistochemistry (IHC)

The separated tumor tissues were fixed in formalin, dehydrated in ascending grades of ethanol, embedded in paraffin, and sectioned at 4 μm thickness. After dewaxing and dehydration, the tissue sections were incubated in 3% hydrogen peroxide for 10 min and microwaved for antigen removal for 15 min. After blocked with bovine serum albumin, the sections were incubated with FANCD2 antibody (ab108928, 1:1000, Abcam) overnight at 4°C, followed by secondary antibody (1:500) at 37°C for another 30 min. Then sections were stained by diaminobenzidine and counterstained by hematoxylin. Finally, all tissue sections were observed under an inverted fluorescence microscope (Olympus, Tokyo, Japan).

### Statistical analysis

Statistical data were presented as the mean ± SD and were analyzed by SPSS 22.0 software. Student’s t-test was used to analyze two-group comparisons. Multiple group comparisons were analyzed using one-way or two-way ANOVA followed Tukey’s multiple comparisons test. P < 0.05 was considered statistically significant.

## Results

### FANCD2 is up-regulated in osteosarcoma cells

**Fig. 1:**
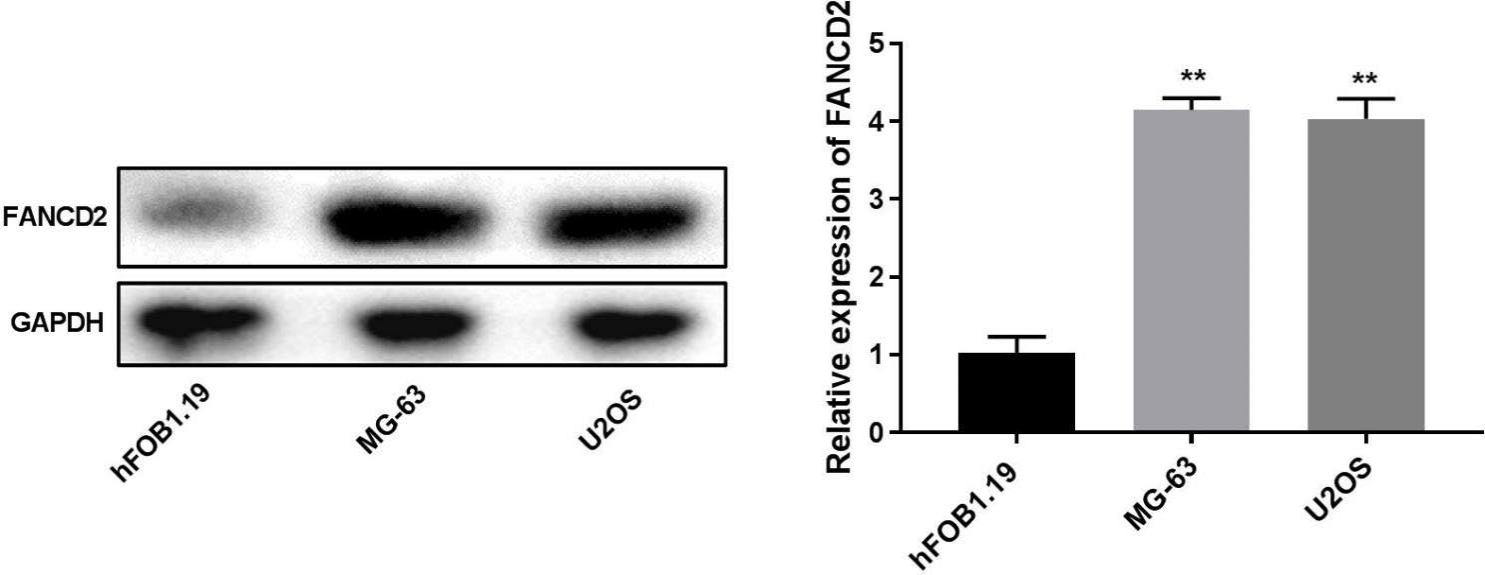
FANCD2 is up-regulated in osteosarcoma cells. Relative protein expression of FANCD2 in osteosarcoma cells was determined by western blot. ^**^P < 0.01 versus hFOB1.19

The levels of FANCD2 were detected in osteosarcoma cells (MG-63 and U2OS cells). Western blot assay indicated that FANCD2 was highly expressed in MG-63 and U2OS cells compared with hFOB1.19 cells (Fig. 1, P < 0.01).

### FANCD2 silencing inhibits cell proliferation, migration, and invasion of osteosarcoma cells

**Fig. 2:**
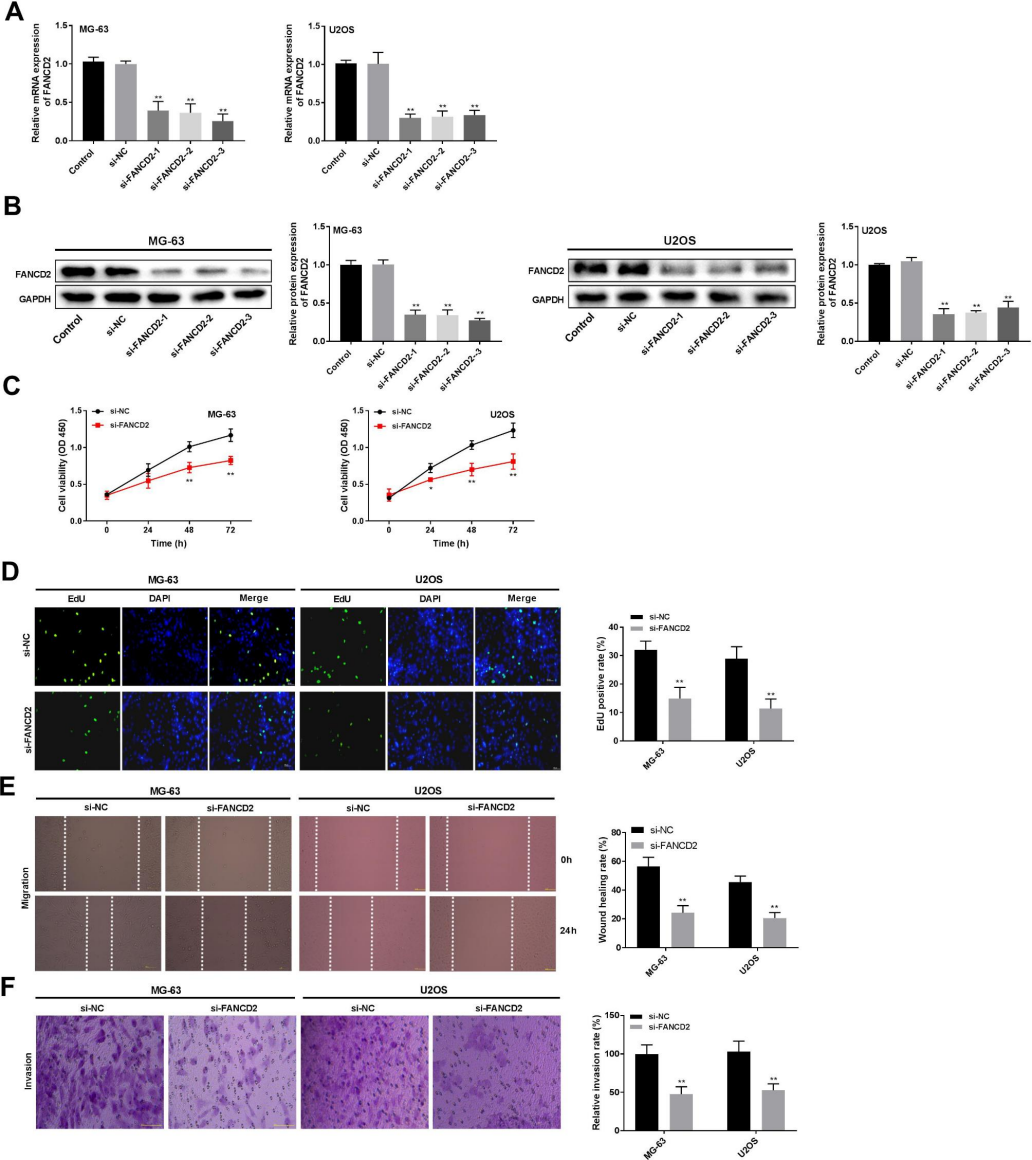
FANCD2 silencing inhibits cell proliferation, migration, and invasion of osteosarcoma cells. (A) FANCD2 mRNA expression was detected by RT-qPCR. (B) FANCD2 protein expression was determined by western blot; the membranes were cut prior to hybridisation with antibodies. (C) Cell viability of MG-63 and U2OS cells was measured by MTT assay. (D) The proliferation of MG-63 and U2OS cells was detected by the EdU detection kit. E-F. The migration and invasion of MG-63 and U2OS cells were determined by wound healing and transwell assay. ^**^P < 0.01 versus si-NC

To investigate the function of FANCD2 in osteosarcoma cells, FANCD2 was knocked down by transfection of si-FANCD2 in MG-63 and U2OS cells. The mRNA and protein expression levels of FANCD2 were both reduced after transfected with si-FANCD2-1, si-FANCD2-2, and si-FANCD2-3 in MG-63 and U2OS cells (Fig. 2A-B, P < 0.01). si-FANCD2-1 was selected for subsequent test experiments. Compared with the si-NC group, the viability of MG-63 and U2OS cells at 48 and 72 h, and the EdU-positive cells were reduced by silencing of FANCD2 (Fig. 2C-D, P < 0.01). Furthermore, compared with the si-NC group, knockout of FANCD2 inhibited migration and invasion of MG-63 and 143B cells (Fig. 2E-F, P < 0.01).

### FANCD2 silencing predisposes ferroptosis of osteosarcoma cells

**Fig. 3:**
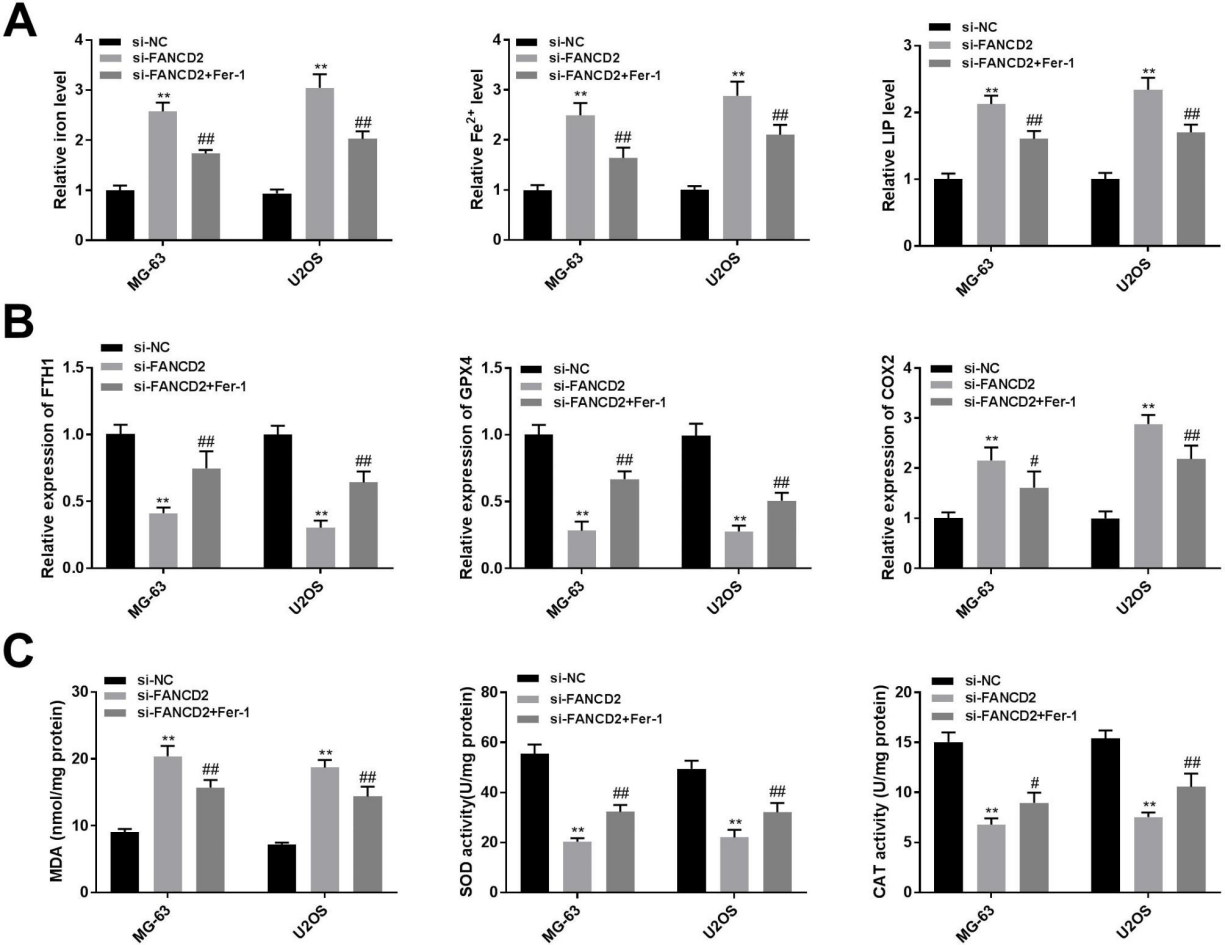
FANCD2 silencing predisposes ferroptosis. (A) The levels of iron, ferrous iron, and LIP were determined by iron detection kits and calcein-acetoxymethyl ester method, respectively. (B) The expression of ferroptosis-related genes (FTH1, GPX4, and COX2) was determined by RT-qPCR. (C) MDA, SOD, and CAT content were determined by the appropriate kits. ^**^P < 0.01 versus si-NC. ^##^P < 0.01 versus si-FANCD2

Ferroptosis regulates the progression of osteosarcoma and play an essential role in osteosarcoma treatment [4]. To evaluate the effects of FANCD2 on ferroptosis in MG-63 and U2OS cells, the ferroptosis-related markers were detected. The si-FANCD2 group has increased levels of iron, Fe^2+^, and LIP in MG-63 and U2OS cells than that in the si-NC group (Fig. 3A, P < 0.01). FTH1, GPX4, and COX2 are associated with the progression of ferroptosis [18, 19]. FANCD2 silencing reduced the levels of FTH1 and GPX4, and up-regulated COX2 in MG-63 and U2OS cells than that in the si-NC group (Fig. 3B, P < 0.01). In addition, lipid peroxidation is an important regulator of ferroptosis [20]. In the si-FANCD2 group, FANCD2 silencing increased MDA level, while reduced the level of SOD and CAT (Fig. 3C, P < 0.01). Notably, the intervention of Fer-1, a ferroptosis inhibitor significantly reversed the promoting effects of si-FANCD2 on ferroptosis, presenting elevated levels of SOD, CAT, FTH1, and GPX4 expression, and decreased levels of iron, Fe^2+^, LIP, MDA, and COX2 expression (Fig. 3A-C, P < 0.01).

### FANCD2 silencing inhibits the JAK2/STAT3 pathway

**Fig. 4:**
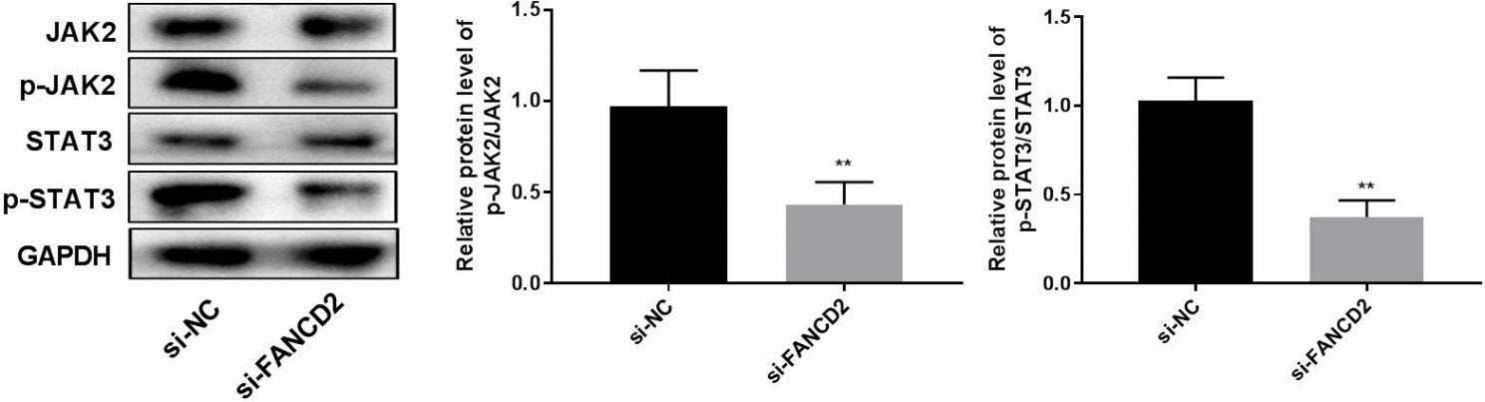
FANCD2 interacts with the JAK2/STAT3 pathway. The expression of JAK, p-JAK, STAT3, and p-STAT3 was determined by western blot. ^**^P < 0.01 versus si-NC

JAK2/STAT3 pathway can regulate tumor occurrence and development [21]. To examine whether JAK2/STAT3 pathway is involved in osteosarcoma development, the expression of JAK2/STAT3 pathway-related genes was detected. As shown in Fig. 4, compared with si-NC group, phosphorylation levels of JAK2 and STAT3 were reduced after FANCD2 knockdown (Fig. 4, P < 0.01).

### Colivelin attenuates the inhibition effects of FANCD2 silencing on malignant phenotype of osteosarcoma cells

**Fig. 5:**
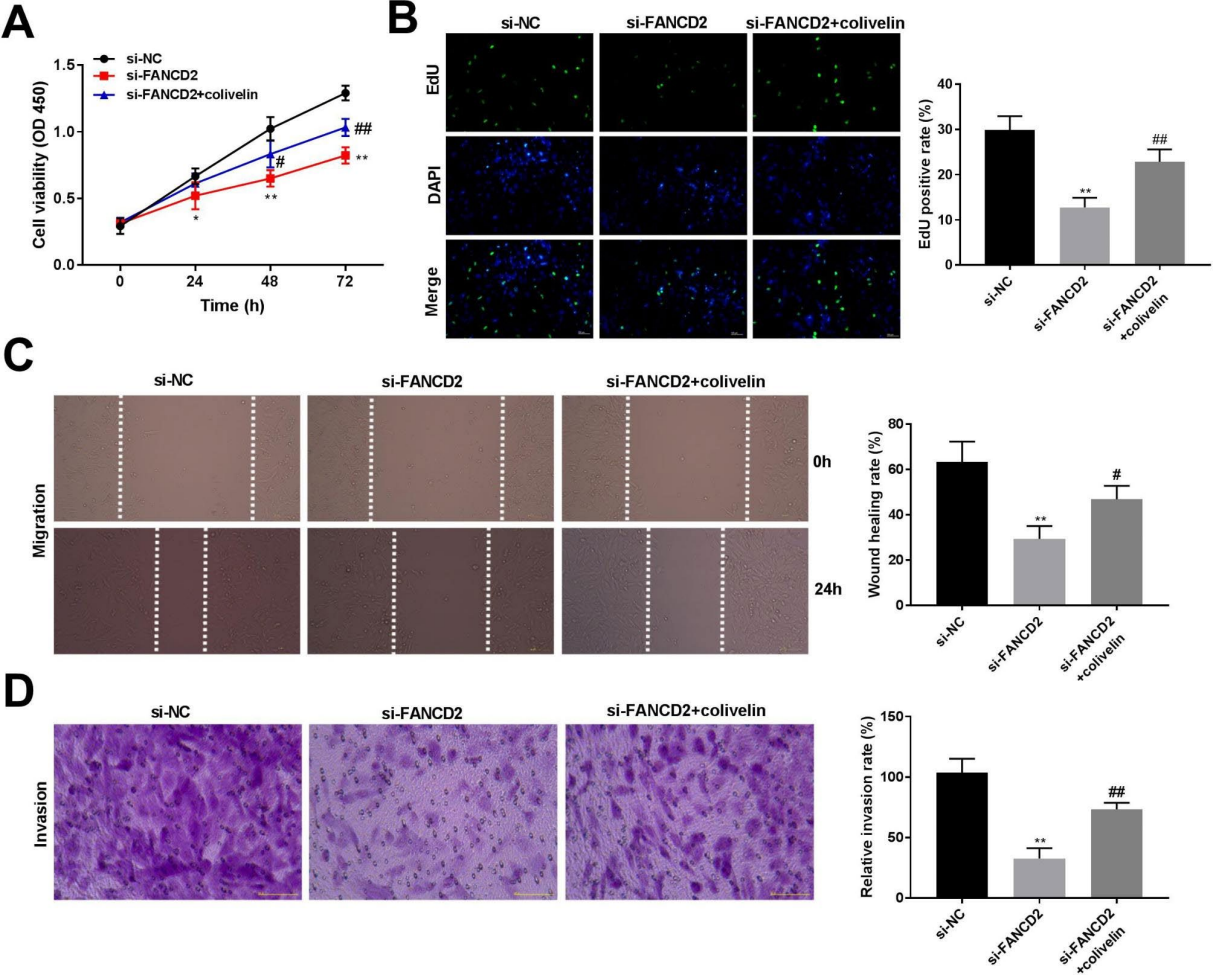
Colivelin attenuates the inhibition effects of FANCD2 silencing on malignant phenotype of osteosarcoma cells. (A) Cell viability of U2OS cells was determined by MTT assay. (B) The proliferation of U2OS cells was detected by the EdU detection kit. C-D. Wound healing and transwell assay were used to analyze U2OS cell migration and invasion. ^*^P < 0.05, ^**^P < 0.01 versus si-NC. ^#^P < 0.05, ^##^P < 0.01 versus si-FANCD2

To explore whether FANCD2 regulates osteosarcoma development by JAK2/STAT3, U2OS cells were treated with STAT3 activator (colivelin, 5 µM). Colivelin weakened the inhibitory effects of FANCD2 silencing on cell viability and proliferation of U2OS cells compared with si-FANCD2 (Fig. 5A-B, P < 0.01). Meanwhile, migration and invasion of U2OS cells were increased in si-FANCD2 + colivelin group compared with si-FANCD2 (Fig. 5C-D).

### Colivelin reverses the promoting effects of FANCD2 silencing on ferroptosis in U2OS cells

**Fig. 6:**
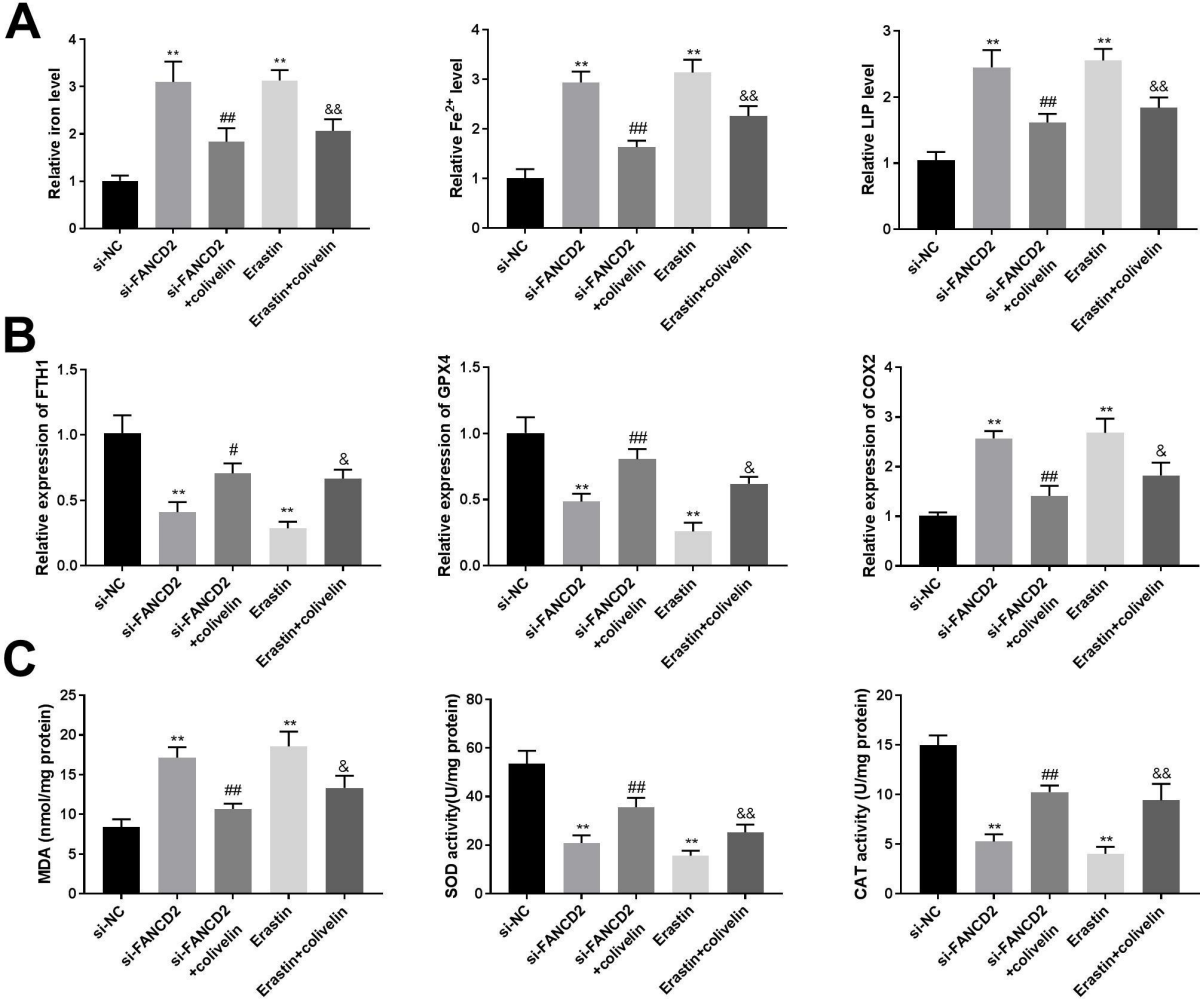
Colivelin reverses the effects of FANCD2 silencing on ferroptosis. (A) The levels of iron, Fe^2+^, and LIP were determined by iron detection kits and calcein-acetoxymethyl ester method. (B) RT-qPCR was used to detect the expression of ferroptosis-related genes. (C) The levels of MDA, SOD, and CAT were detected by the appropriate kits. ^**^P < 0.01 versus si-NC. ^#^P < 0.05, ^##^P < 0.01 versus si-FANCD2. ^&^P < 0.05, ^&&^P < 0.01 versus Erastin

To determine the role of JAK2/STAT3 pathway on ferroptosis, cells were treated with colivelin and examined the effect on ferroptosis. Compared with the si-FANCD2 group, the levels of iron, Fe^2+^, and LIP in U2OS cells were decreased in the si-FANCD2 + colivelin group (Fig. 6A, P < 0.01). Compared with the si-FANCD2 group, colivelin reversed the effects of FANCD2 silencing on genes related to ferroptosis, including FTH1, GPX4, and COX2 (Fig. 6B, P < 0.01). In addition, colivelin reduced MDA content, while increased SOD and CAT levels, which were opposite to the si-FANCD2 group (Fig. 6C, P < 0.01). Furthermore, Erastin, a ferroptosis inducer exhibited consistent effects with si-FANCD2 on elevating iron, Fe^2+^, LIP, MDA, and COX2, and on decreasing SOD, CAT, FTH1, and GPX4 in U2OS cells (Fig. 6A-C, P < 0.05). However, the inducing effects of Erastin on ferroptosis was significantly reversed by the treatment of colivelin (Fig. 6A-C, P < 0.05).

### Silencing of FANCD2 inhibits tumor growth in vivo

**Fig. 7:**
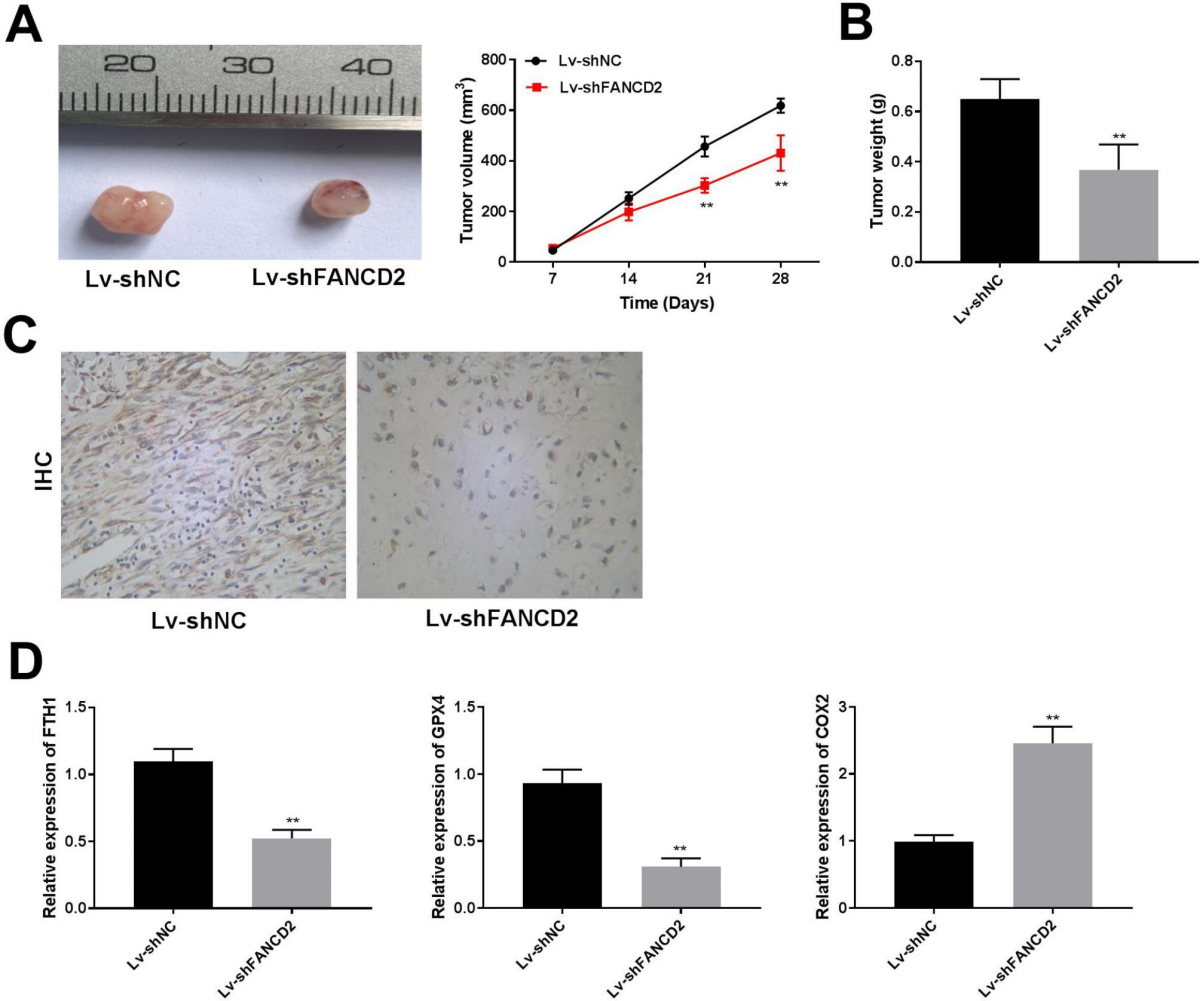
FANCD2 silencing inhibits tumor growth. (A) Tumor tissue images and tumor volume were measured in different groups. (B) Tumor weight was assessed in different groups. ^**^P < 0.01 versus Lv-sh-NC. (C) IHC staining of FANCD2 expression in tumor tissues of Lv-shNC and Lv-shFANCD2 groups. (D) The expression of ferroptosis-related genes in tumor tissues was detected by RT-qPCR

The role of FANCD2 was further explored in xenograft tumor mice models. Compared with the Lv-shNC group, the Lv-shFANCD2 group has a lower tumor volume and weight (Fig. 7A-B, P < 0.01). Besides, IHC assay confirmed the down-regulation of FANCD2 in tumor tissues of the Lv-shFANCD2 group than that in the Lv-shNC group (Fig. 7C). Furthermore, FANCD2 knockdown reduced the expression of FTH1, while up-regulated GPX4 and COX2 compared with the Lv-shNC group (Fig. 7D). The results suggested that FANCD2 silencing contributed to suppressing tumor growth and regulated ferroptosis-related genes.

## Discussion

Osteosarcoma has a high propensity for local invasion and metastasis, although surgery and chemotherapy have greatly improved the prognosis of patients with osteosarcoma, the prognosis is still unsatisfactory [22]. Hence, it is urgent to explore the potential mechanisms involving osteosarcoma progression to seek possible targets. In our study, these results indicated that FANCD2 silencing inhibited osteosarcoma cells and tumor growth, as well as promoted ferroptosis by activating JAK2/STAT3 pathway.

At present, some previous evidence has demonstrated the function of FANCD2 in promoting tumor development, such as nasopharyngeal carcinoma [23], melanoma [24], and hepatocellular carcinoma [25]. For example, FANCD2 silencing significantly inhibits cell proliferation, promotes apoptosis of CNE-2 cells, and the silencing of FANCD2 slows tumor growth in the xenograft tumor models of nasopharyngeal carcinoma [23]. Melanoma cells have a higher FANCD2 expression, FANCD2 promotes the proliferation and survival of melanoma cells, and may serve as a biomarker for melanoma [24]. FANCD2 is up-regulated in hepatocellular carcinoma tissues, elevated FANCD2 expression is associated with poorer prognoses, larger tumor size, and invasive phenotypes, as well as knockdown of FANCD2 attenuates hepatocellular carcinoma cell proliferation and invasion [25]. Similar to previous research, in the present study, FANCD2 expression was obviously up-regulated in osteosarcoma cells. In addition, knockdown of FANCD2 decreased cell viability and proliferation, inhibited the ability of invasion and migration of osteosarcoma cells, and suppressed tumor growth in xenograft models. Taken together, FANCD2 silencing inhibited the malignant phenotype of osteosarcoma. Consistent with our studies, Xia et al. have reported that silencing of FANCD2 attenuates cell cycle and proliferation, and promotes cell apoptosis in MG-63 cells [6].

Ferroptosis is vital to the regulation of osteosarcoma progression, and modulating ferroptosis may be a potential treatment strategy for osteosarcoma therapy [4, 26]. For instance, bavachin induces ferroptosis in osteosarcoma cells by down-regulating STAT3 and GPX4, and increasing intracellular Fe^2+^ levels, thereby exerting anti-tumor effects on osteosarcoma [12]. Shi et al. have found that Tirapazamine could induce ferroptosis by inhibiting SLC7A11 to inhibit the proliferation and migration of osteosarcoma cells [27]. Furthermore, high expression of FANCD2 can promote temozolomide resistance in glioblastoma cells by attenuating ferroptosis, while FANCD2 knockdown increases the levels of ROS and inhibits cell survival [7]. Based on previous studies, we speculated that FANCD2 may regulate osteosarcoma progression by mediating ferroptosis. In this research, we detected the role of FANCD2 on ferroptosis of osteosarcoma cells, FANCD2 knockdown reduced the levels of FTH1 and GPX4, and elevated COX2, increased the levels of iron, LIP, Fe^2+^, and promoted lipid peroxidation. In addition, Fer-1, a ferroptosis inhibitor reversed the promoting effects of FANCD2 knockdown on ferroptosis. Taken together, we suggested that FANCD2 knockout contributed to promoted ferroptosis of osteosarcoma cells, thereby inhibiting the progression of osteosarcoma, targeting ferroptosis through FANCD2 may be a potential strategy.

STAT3 can regulate cell apoptosis, proliferation, and tumor invasion [28]. The JAK2/STAT3 axis is activated in human GC tissues, and JAK2/STAT3 signaling activation exerts a role in tumor growth and metastasis [29]. Panaxadiol inhibits proliferation, and induced apoptosis of pancreatic cancer cells, and suppresses the growth of xenograft models by suppressing the JAK2/STAT3 pathway [30]. In addition, the activation of JAK2/STAT3 pathway regulates malignant behaviors of glioma cells, including the proliferation and invasion of glioma cells [31]. A recent study showed that miR-19a can inhibit the JAK2/STAT3 pathway, thereby inhibiting cell proliferation and promoting apoptosis of SaOS-2 cells [32]. In addition, some evidence has investigated that JAK2/STAT3 pathway is involved in the regulation of ferroptosis, such as gastric cancer and head and neck squamous cell carcinoma [33, 34]. Meanwhile, the reduction of STAT3 and GPX4 leads to increased ferroptosis in osteosarcoma cells [35]. Based on previous research, we speculated that FANCD2 interacts with JAK2/STAT3 pathway to mediate osteosarcoma development. In the present study, colivelin reduced the inhibitory effects of FANCD2 up-regulation and also erastin (a ferroptosis inducer) on cell viability, invasion, migration, and inhibited ferroptosis of osteosarcoma cells. Taken together, we indicated that FANCD2 may mediate ferroptosis in osteosarcoma by JAK2/STAT3 pathway to regulate tumor cell growth.

However, there are still some limitations in this paper. Firstly, the regulatory mechanism of FANCD2 relating with the JAK2/STAT3 pathway was only confirmed in one cell line. Secondly, whether FANCD2 can be used as a prognosis marker for osteosarcoma needs to be demonstrated in clinical samples. Finally, more deep action mechanisms of FANCD2 in osteosarcoma still need to be further studied.

In summary, in this research, we investigated the function of FANCD2 on osteosarcoma cells. These results demonstrated that FANCD2 could inhibit cell viability, invasion, and migration of osteosarcoma cells, and suppress tumor growth in vivo. Meanwhile, FANCD2 knockdown predisposed osteosarcoma cells to ferroptosis by regulating the JAK2/STAT3 pathway. Our findings may provide novel light on possible therapeutic strategies for the treatment of osteosarcoma.

## Electronic supplementary material

Below is the link to the electronic supplementary material.

Supplementary Material 1

## Data Availability

The datasets generated and/or analysed during the current study are not publicly available due [Internal policy of Minhang Hospital, Fudan University] but are available from the corresponding author on reasonable request.
