# Supplementary material for: FANCD2 inhibits ferroptosis by regulating the JAK2/STAT3 pathway in osteosarcoma
Source: BMC Cancer. 2023 Feb 22;23:179. doi: 10.1186/s12885-023-10626-7 (PMC9945409; doi:10.1186/s12885-023-10626-7)
Supplement: Supplementary file 1 — Supplementary Material 1 [file 12885_2023_10626_MOESM1_ESM.pdf]

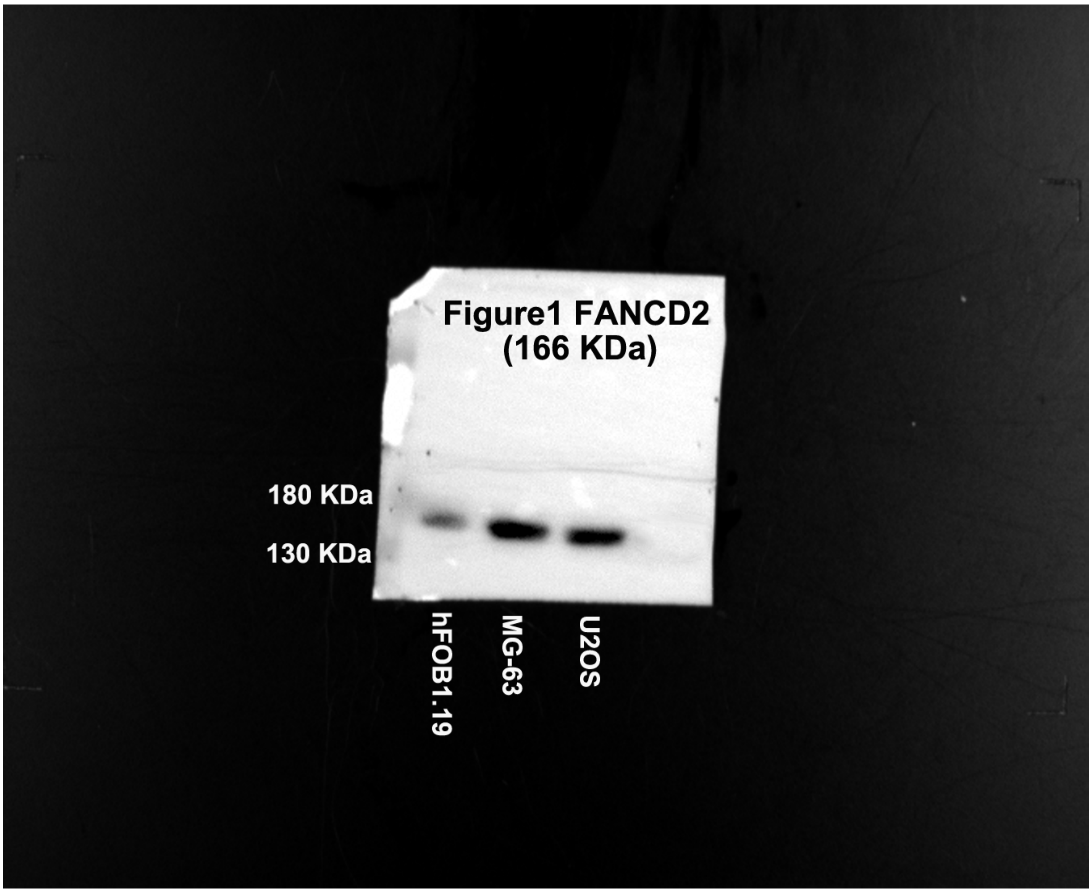
Figure1 FANCD2
(166 KDa)
180 KDa
130 KDa
hFOB1.19
MG-63
U2OS

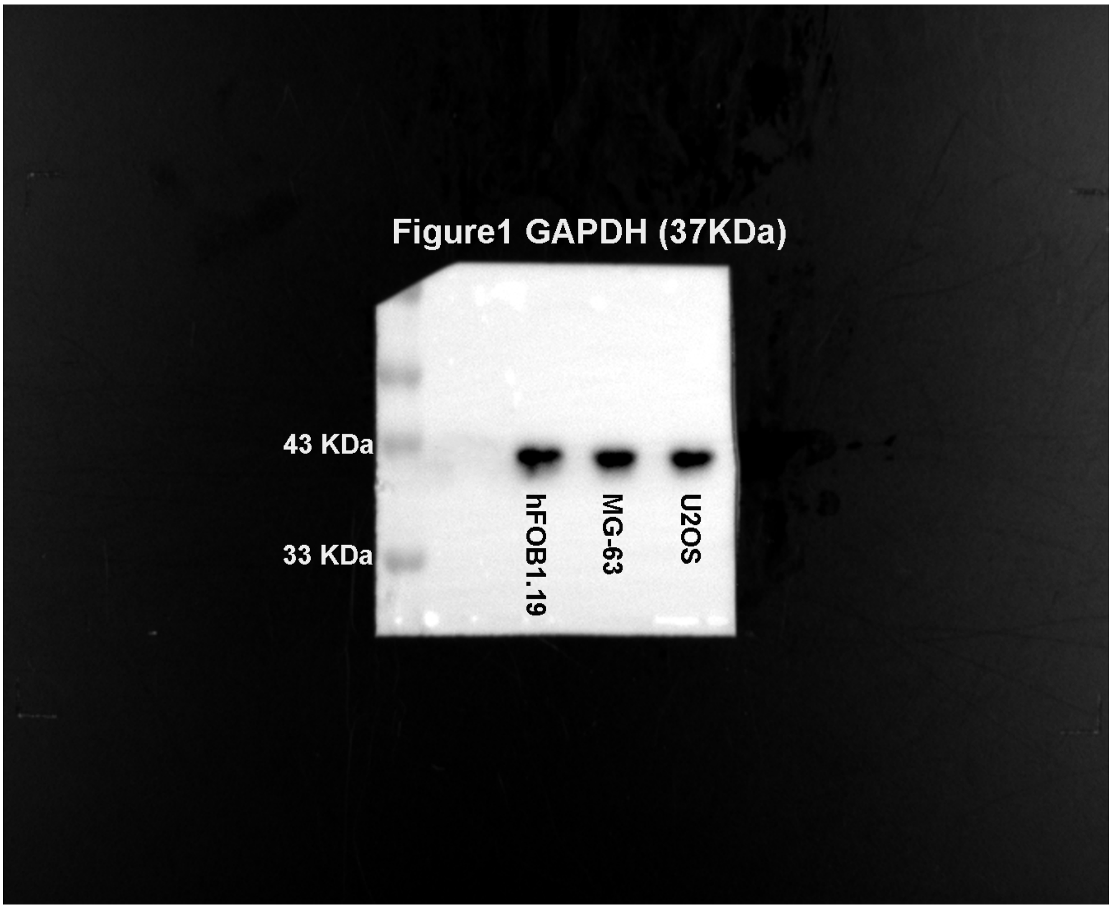

Figure1 GAPDH (37KDa)
43 KDa
33 KDa
hFOB1.19
MG-63
U2OS

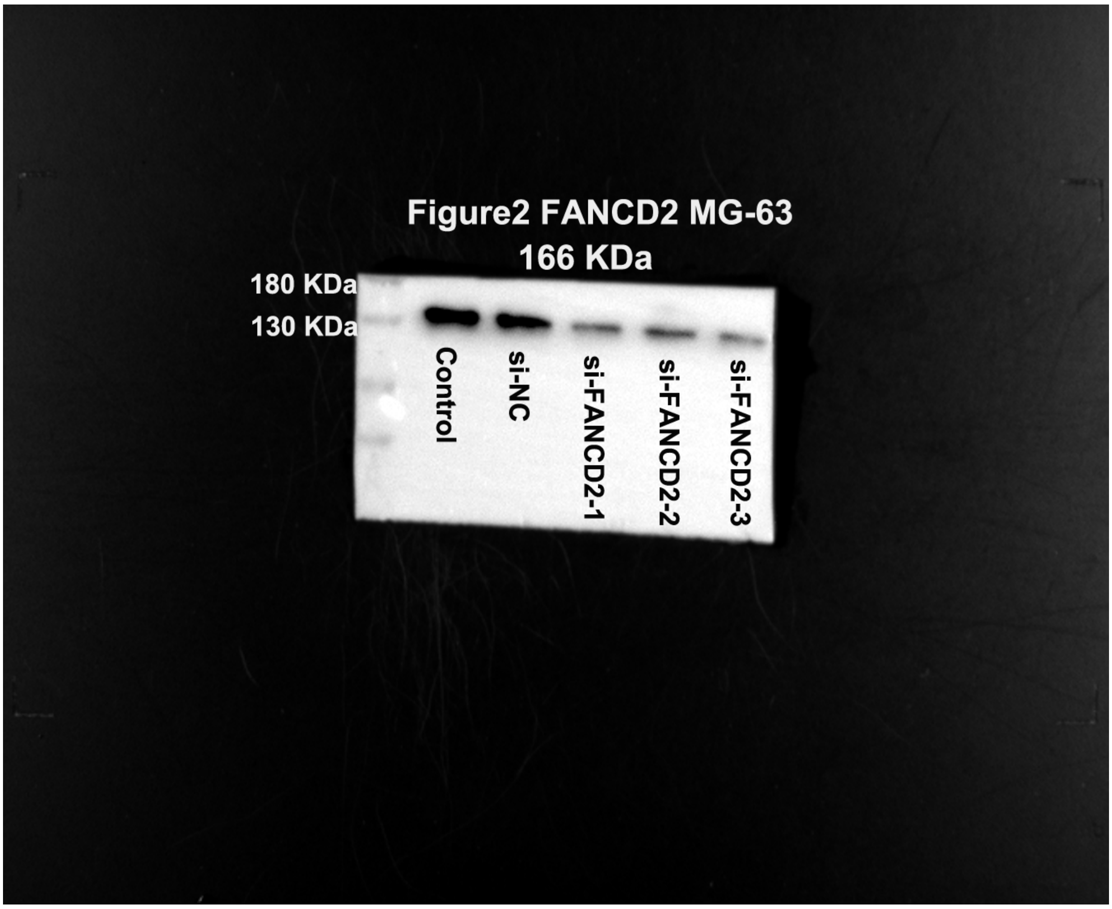
Figure2 FANCD2 MG-63
166 KDa
180 KDa
130 KDa
Control
si-NC
si-FANCD2-1
si-FANCD2-2
si-FANCD2-3

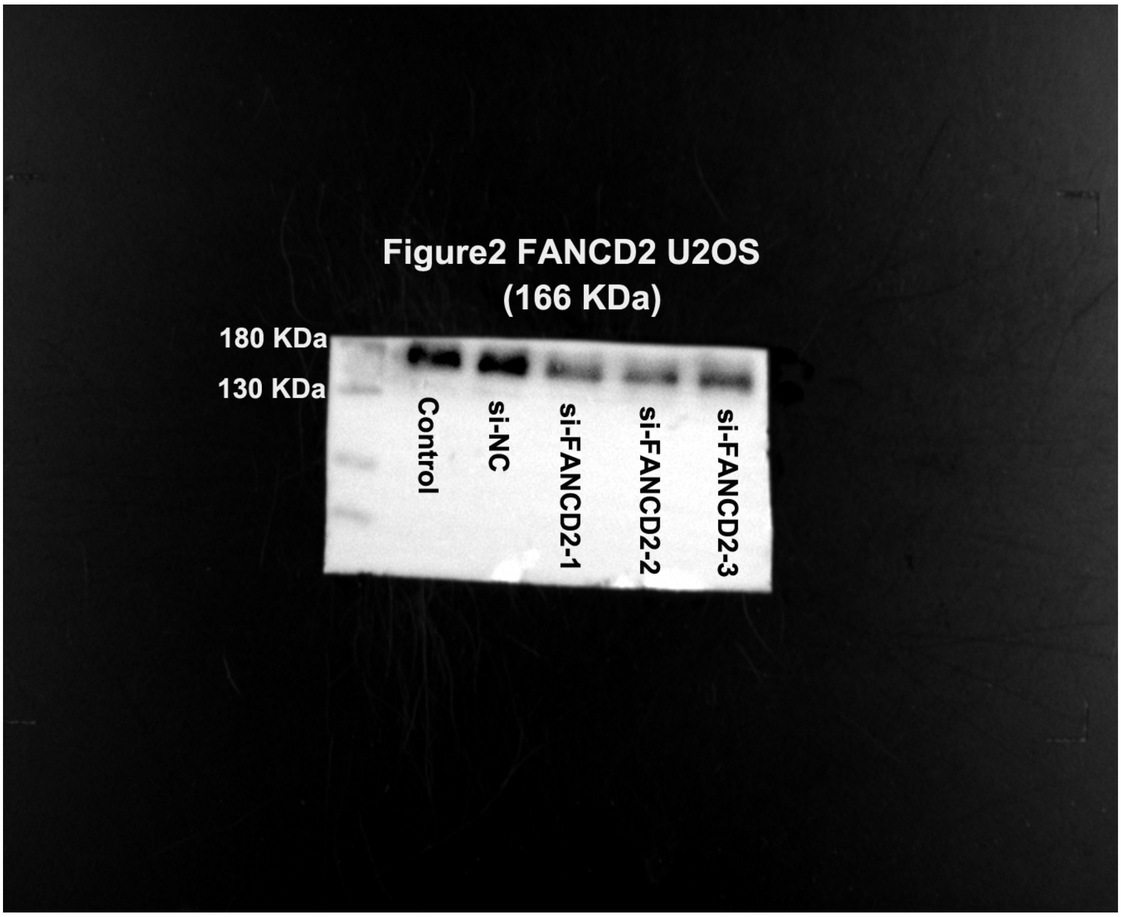
Figure2 FANCD2 U2OS
(166 KDa)
180 KDa
130 KDa
Control
si-NC
si-FANCD2-1
si-FANCD2-2
si-FANCD2-3

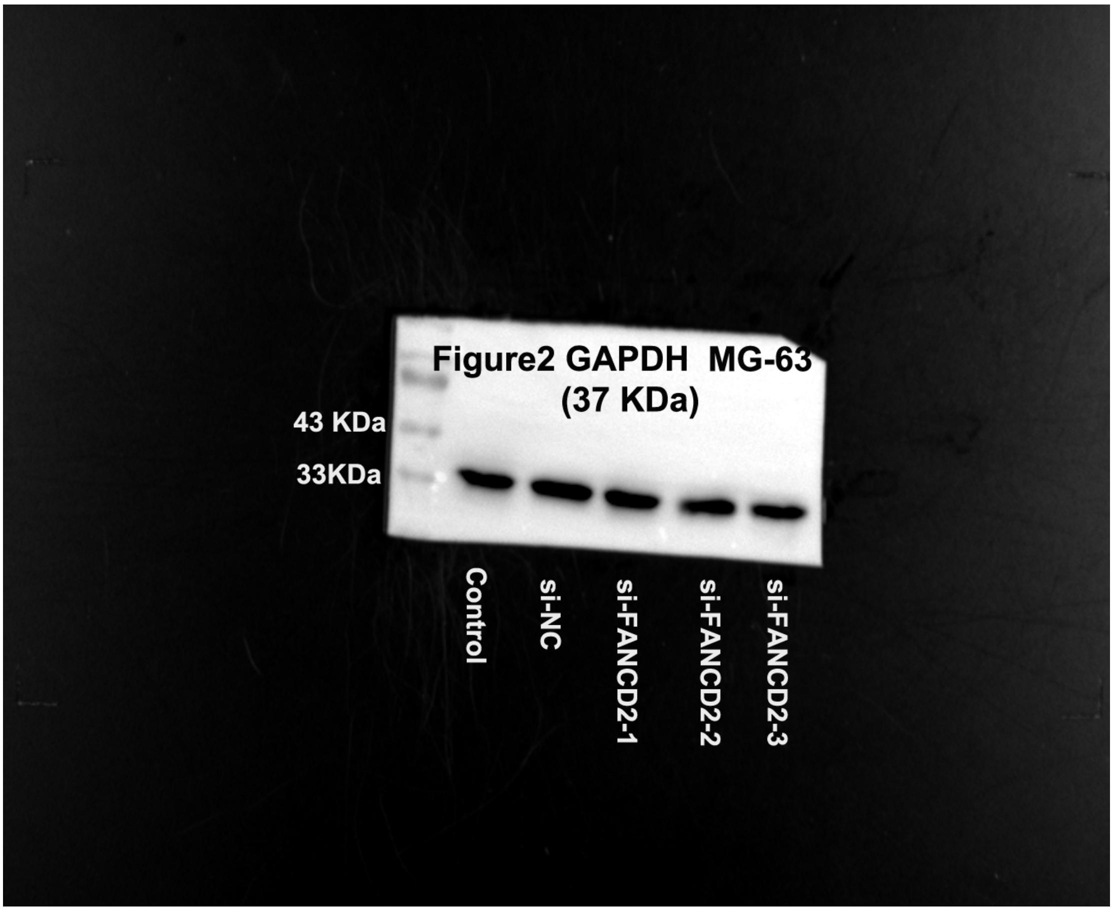
Figure2 GAPDH MG-63
(37 KDa)
43 KDa
33KDa
Control
si-NC
si-FANCD2-1
si-FANCD2-2
si-FANCD2-3

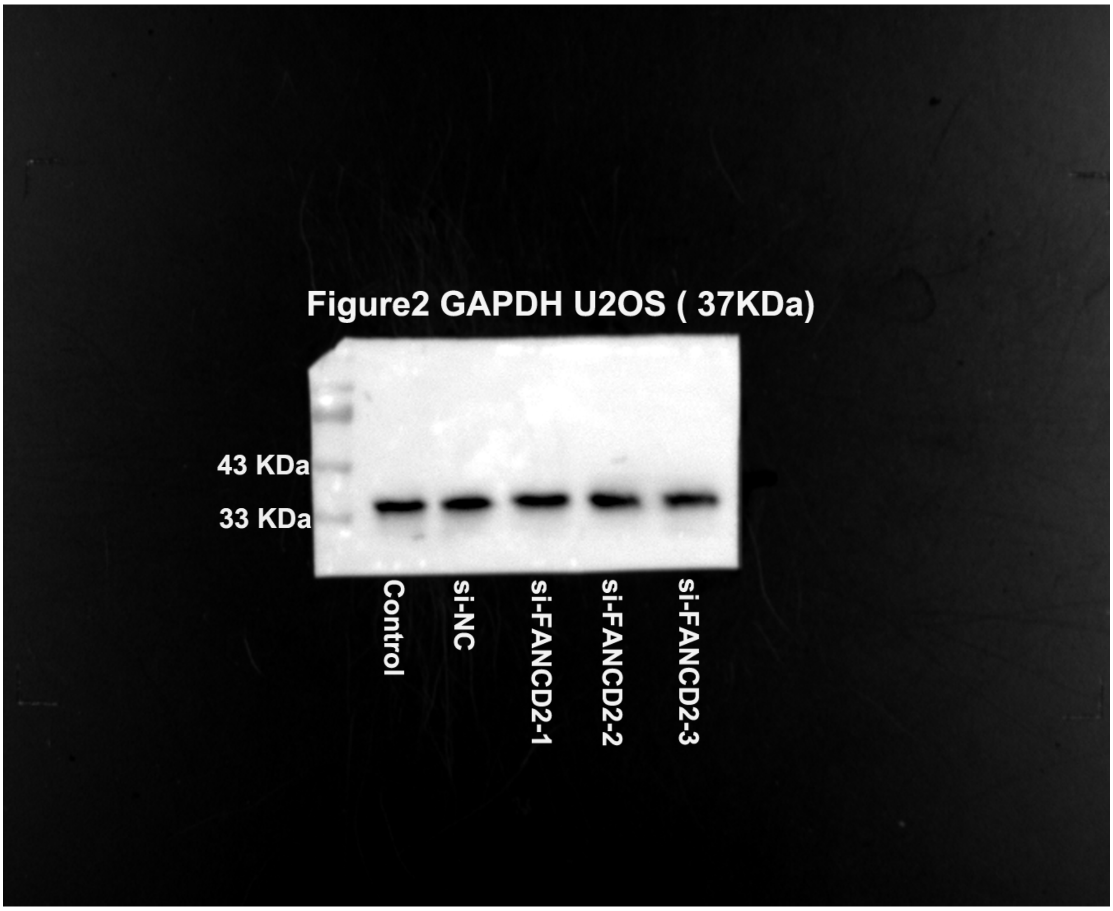
Figure2 GAPDH U2OS (37KDa)
43 KDa
33 KDa
Control
si-NC
si-FANCD2-1
si-FANCD2-2
si-FANCD2-3

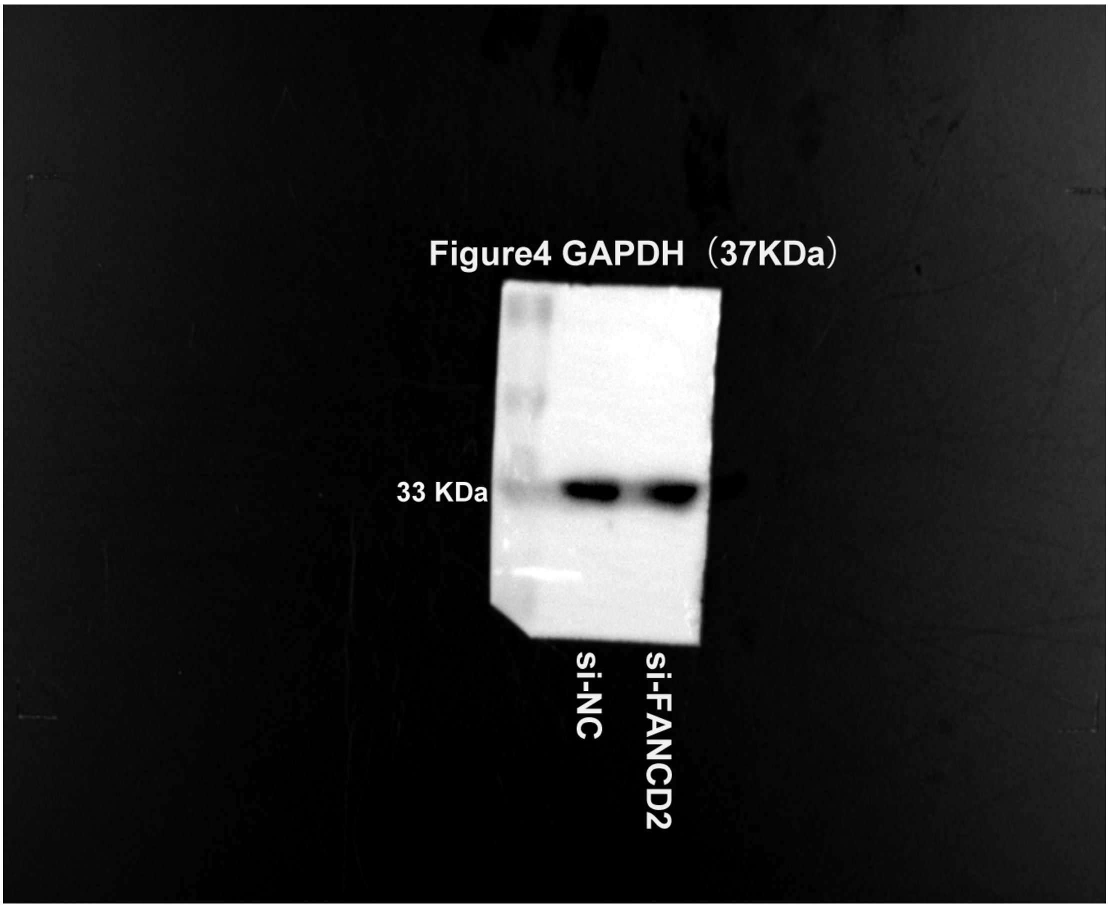
Figure4 GAPDH（37KDa）
33 KDa
si-NC
si-FANCD2

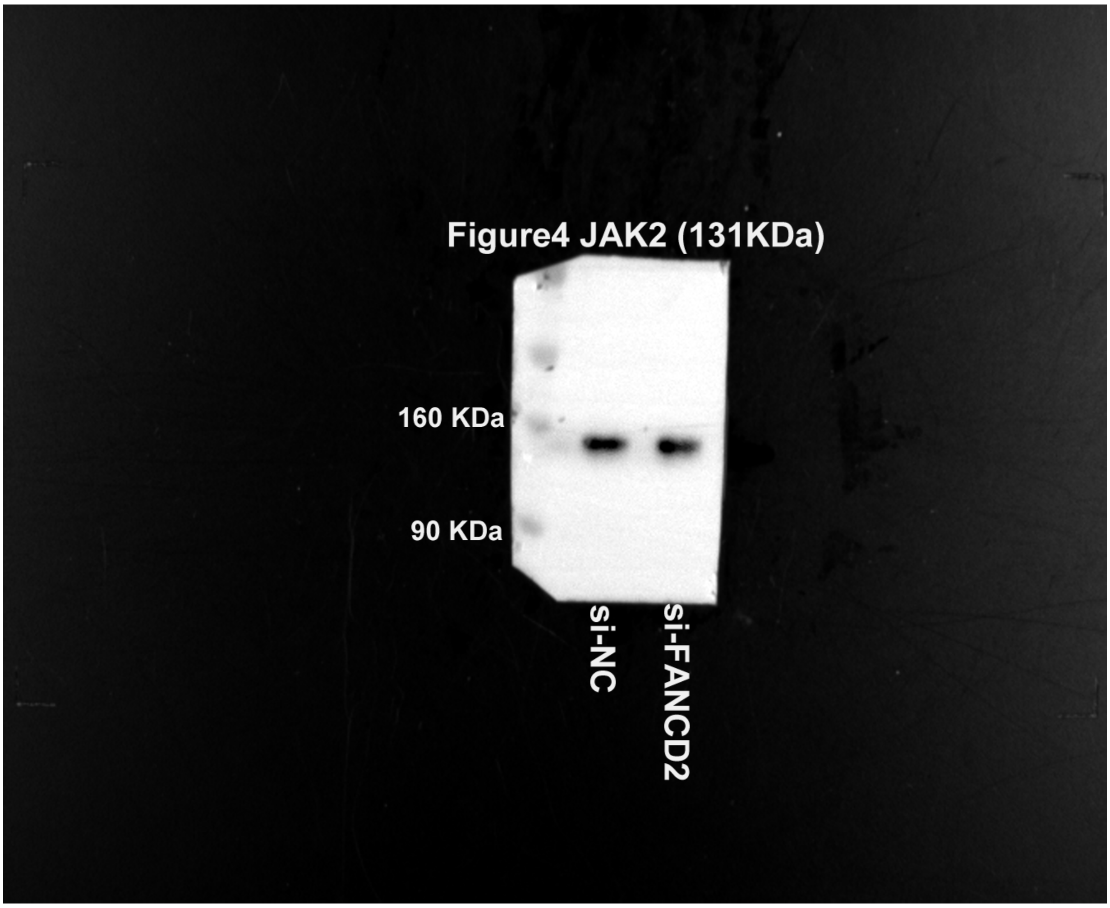
Figure4 JAK2 (131KDa)
160 KDa
90 KDa
si-NC
si-FANCD2

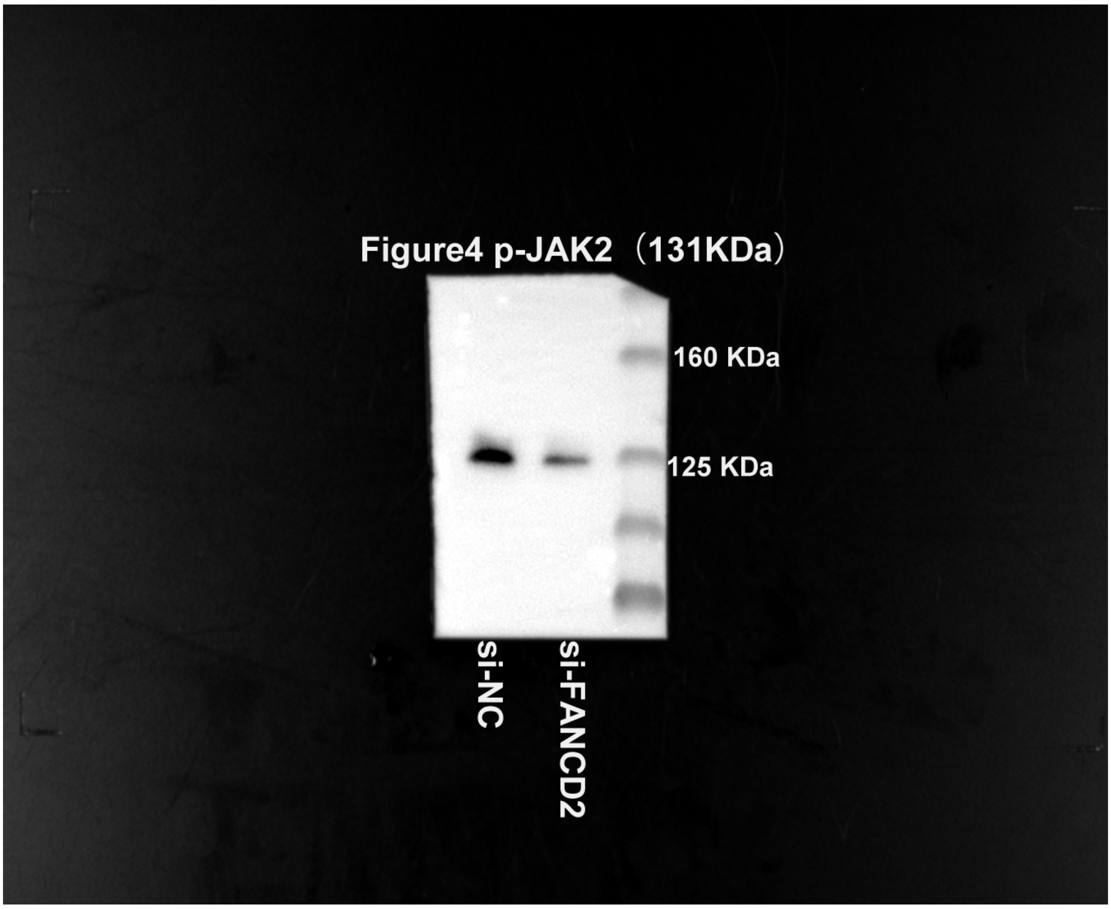
Figure4 p-JAK2（131KDa）
160 KDa
125 KDa
si-NC
si-FANCD2

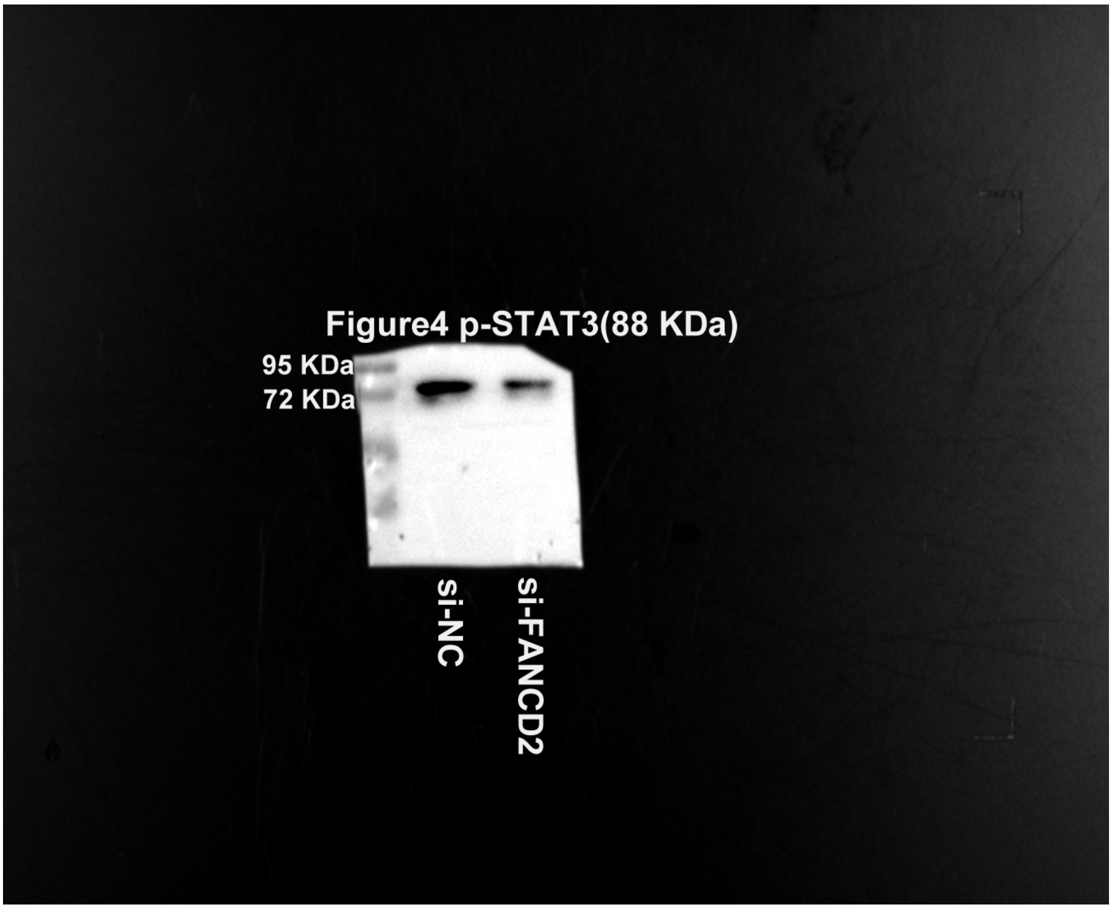
Figure4 p-STAT3(88 KDa)
95 KDa
72 KDa
si-NC
si-FANCD2

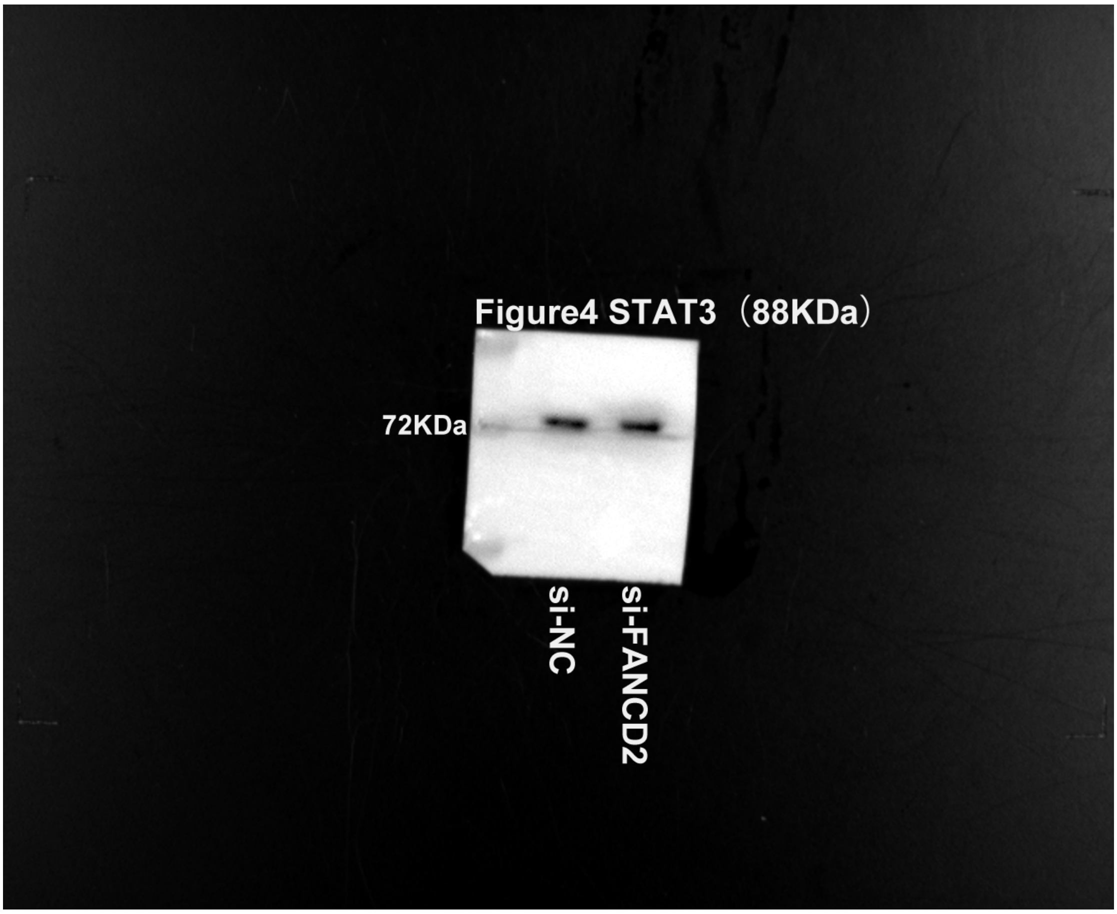
Figure4 STAT3（88KDa）
72KDa
si-NC
si-FANCD2
